# *N*-Arylacetamide derivatives of methyl 1,2-benzothiazine-3-carboxylate as potential drug candidates for urease inhibition

**DOI:** 10.1098/rsos.230104

**Published:** 2023-04-05

**Authors:** Sajila Hina, Sumera Zaib, Maliha Uroos, Muhammad Zia-ur-Rehman, Rubina Munir, Huma Riaz, Quratulain Syed, Syed Hussain Imam Abidi

**Affiliations:** ^1^ Centre for Research in Ionic Liquids, School of Chemistry, University of the Punjab, Quaid e Azam Campus, Lahore 54590, Pakistan; ^2^ Applied Chemistry Research Centre, PCSIR Laboratories Complex, Lahore 54600, Pakistan; ^3^ Department of Basic and Applied Chemistry, Faculty of Science and Technology, University of Central Punjab, Lahore 54590, Pakistan; ^4^ Department of Chemistry, Kinnaird College for Women, Lahore 54000, Pakistan; ^5^ Pakistan Council of Scientific and Industrial Research, 01-Constitution Avenue, G-5/2, Islamabad 44050, Pakistan

**Keywords:** benzothiazine, synthesis, urease, thiourea, docking, ureolytic

## Abstract

Urease enzyme is an infectious factor that provokes the growth and colonization of virulence pathogenic bacteria in humans. To overcome the deleterious effects of bacterial infections, inhibition of urease enzyme is one of the promising approaches. The current study is designed to synthesize new 1,2-benzothiazine-*N*-arylacetamide derivatives **5**(**a-n**) that can effectively provide a new drug candidate to avoid bacterial infections by urease inhibition. After structural elucidation by FT-IR, proton and carbon-13 NMR and mass spectroscopy, the synthesized compounds **5**(**a-n**) were investigated to evaluate their inhibitory potential against urease enzyme. *In vitro* analysis against positive control of thiourea indicated that all the synthesized compounds have strong inhibitory strengths as compared to the reference drug. Compound **5k**, being the most potent inhibitor, strongly inhibited the urease enzymes and revealed an IC_50_ value of 9.8 ± 0.023 µM when compared with the IC_50_ of thiourea (22.3 ± 0.031 µM)—a far more robust inhibitory potential. Docking studies of **5k** within the urease active site revealed various significant interactions such as H-bond, π-alkyl with amino acid residues like Val744, Lys716, Ala16, Glu7452, Ala37 and Asp730.

## 1. Introduction

Enzyme inhibition remains the most attractive research domain in drug design as most of the natural physiological pathways involve a number of enzymes. Urease (EC3.5.1.5; urea amidohydrolase) enzyme having nickel in its core structure catalyses urea dissociation into ammonia and carbamate approximately a hundred trillion times faster than un-catalysed reaction [1]. The family of urease enzyme is common in a large number of prokaryotes and eukaryotes including plants and fungi [2]. Urease has become the most significant target for drug development against pathogens like *Helicobacter pylori* (*H. pylori*), a common cause of stomach-related ailments [3,4]. Production of ammonia enhances the pH, which creates an environment favourable for the progression of *H. pylori* in the stomach [5]. Consequently, urease activity is one of the critical stimuli for various pathogenic gastrointestinal conditions in humans and animals [6]. Thus, the inhibition of urease enzymes is conceivably good for hindering the injurious effects of urinary bacterial diseases in humans [7].

Over the past few years, different anti-urease agents have been documented due to their exceptional inhibitory potential. There are different types of effective urease inhibitors such as hydroxamic acid derivatives [8], hydrazides [9], semicarbazides [10–12], flavonoid glycoside [13], palmatine [14], epiberberine [15], Schiff bases [16], benzimidazoles [17], triazoles or oxadiazoles [18], phosphoramidates [19], boric and boronic acids [20], heavy metal ions [21], thiourea [22] and (thio)barbituric acid derivatives [23,24]. Fluoride ion as a non-competitive inhibitor is capable of inactivating the enzyme [25]. The importance of metallic ligands, hydrogen bonding and hydrophobic moieties as vital medicinal features for urease inhibitors has been explored in many literature studies [26]. Quinazolinones and their derivatives are the heterocyclic systems abundantly used in medicinal chemistry due to their functions such as anti-urease activities [27]. Nowadays, urease inhibitors have become the most vital anti-ulcer drugs that manage the deleterious effects of bacterial infections in humans and animals; however, these are accompanied by side effects and drug-resistance. Owing to a rise in the drug-resistance and toxic side effects of the available medication for urease inhibition, there is a need to prepare new, more potent but less toxic urease inhibitors for the better treatment of gastrointestinal disorders.

**Figure 1.**
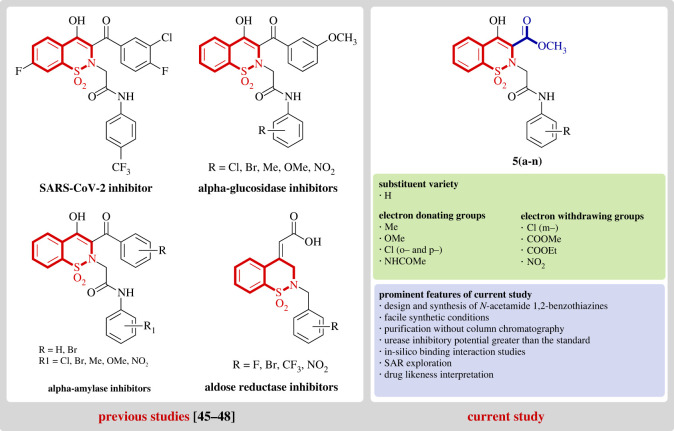
Rationale of current work.

1,2-benzothiazine 1,1-dioxides are versatile pharmacophores which are widely explored for their potent biological activities and hence are considered molecules of great interest [28]. They are well-known non-steroidal anti-inflammatory drugs (NSAIDs) commercially available as oxicams [29]. Studies reveal that 1,2-benzothiazines exhibit wide pharmacological activities such as anti-inflammatory [30], calpain I inhibitors [31], anti-cancer [32], anti-microbial [33], analgesic [34], MAPK inhibitors [35], MAO inhibition [36], anti-arthritic [37], anti-oxidant [38], anti-viral [39,40], anti-diabetic [41], anti-convulsant [42] and anti-malarial [43]. Other applications include as herbicides and as dyestuff in industry [44]. Most of these compounds constitute the *N*-alkyl/acyl 1,2-benzothiazine framework. Similarly, 1,2-benzothiazine-*N*-arylacetamides have also been reported to exhibit pharmacological activities. For example, molecules containing the *N*-arylacetamides functional group have recently been explored as potent SARS-CoV-2 inhibitors (IC_50_ = 0.88 µM) [45]. Similarly, the role of related compounds has been established for inhibition of various enzymes such as aldose reductase, α-glucosidase, α-amylase and human 11β-hydroxysteroid dehydrogenase type 1 (11β-HSD1) [46–48] (figure 1).

Interestingly, despite their rich biological profile benzothiazine derivatives have not been profoundly tested against urease enzyme. This inspired us to assess the urease inhibitory potential of benzothiazine derivatives. In this regard, a renowned anti-urease pharmacophore phenylacetamide was incorporated into the benzothiazine ring. Hence, continuing our previous work to explore new biologically active compounds [49–54], we herein present the synthesis and evaluation of the pharmacological potential of a series of new methyl 1,2-benzothiazine-*N*-arylacetamide derivatives against urease enzyme. The prepared compounds were subjected to characterization through nuclear magnetic resonance spectroscopy (proton and carbon) as well as mass spectrometry. Moreover, urease inhibition activity for the synthesized compounds was also evaluated.

## 2. Results and discussion

### 2.1. Chemistry

**Scheme 1.**
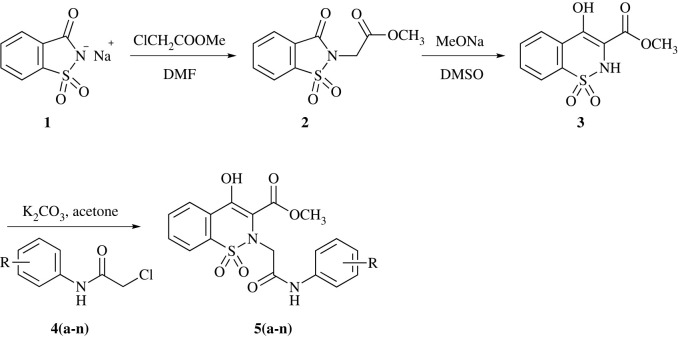
Synthetic route for methyl 1,2-benzothiazine-*N*-arylacetamides **5**(**a-n**).

The schematic synthetic pathway for titled compounds is illustrated in scheme 1. *N*-Alkylation of sodium saccharin under anhydrous conditions gave benzisothiazole substituted ester **2** which underwent ring expansion to yield 1,2-benzothiazine framework **3** [55]. Further *N*-alkylation of benzothiazine bearing ester **3** was carried out by reacting it with α-chloroacetanilides **4** to achieve the desired compounds **5**(**a-n**) in reasonable yields. All the prepared derivatives were characterized by nuclear magnetic resonance spectroscopy (proton and carbon) as well as mass spectrometry.

Proton NMR spectra of all the compounds contain methylene and NH protons which appeared as distinct singlets ranging between 4.34–4.56 and 9.32–10.98 ppm, respectively. The methoxy and enolic -OH protons exhibited singlet peaks at 3.91 ppm and 11.09–11.83 ppm, respectively. Aromatic protons appeared between 6.79 ppm and 8.17 ppm depending upon the extent of deshielding. In ^13^C NMR spectra of the synthesized derivatives, methoxy and methylene carbon atoms showed up around 53.5 ppm and 53.0 ppm respectively while carbonyl carbon appeared as the most deshielded signal at around 168.7 ppm. Signals for aromatic carbon atoms were detected between 108 ppm and 167 ppm. Similarly, in mass spectra, the observed molecular ion peaks were in accordance with the calculated molecular masses.

### 2.2. In *vitro* inhibition and SAR analysis (structure activity relationship)

The synthesized compounds **5**(**a-n**) were evaluated for their inhibitory potential against urease while using thiourea as a positive control (IC_50_ value = 22.3 ± 0.031 µM). The experimental data are outlined in table 1. A general observation of all synthetic analogues was that they had varying degrees of urease inhibitory activity and were remarkably potent. The synthesized compounds showed inhibition in a range of 9.8–20.7 µM. The *in vitro* analysis exhibited that all the synthesized derivatives were exceptionally active and potent inhibitors of urease; even the least active compound of the series **5i** (IC_50_ = 20.7 ± 0.25 µM) revealed better inhibitory potential than the positive control (thiourea) (22.3 ± 0.031 µM). Hence, we analysed a diversification of structural features and influence of different electron donating and withdrawing groups present in the active *N*-phenylacetamide-substituted benzothiazine carboxylate framework. Compound **5k** was selected to be the most potent and effective inhibitor having a IC_50_ value of 9.8 ± 0.023 µM with a twofold stronger inhibitory efficacy than thiourea. The effective structural feature of the most active inhibitor comprised an *N*-phenylacetamide ring bearing a substituent of empirical ethoxy carbonyl at *para*-position. The unsubstituted derivative **5a** exhibited better inhibitory potential against urease (IC_50_ = 10.1 ± 0.90 µM) as compared to urease. A notable decline in the activity was observed when the 2-carboxymethyl group was introduced in the phenylacetamide ring in **5d** (IC_50_ = 14.1 ±0.12 µM). Consequently, substituting the acetamido group at *para*-position on the afore-mentioned ring in **5l**, an IC_50_ value of 12.6 ± 0.10 µM was observed, suggesting the impact of substituents on the efficacy profile of the synthesized analogues. The presence of the halogen (chloro) group as a substituent on different positions of the phenylacetamide ring resulted in moderate inhibitory potentials of **5i** (20.7 ± 0.25 µM), **5m** (14 ± 0.15 µM) and **5n** 13 ± 0.10 µM.

**Table 1.** Urease inhibitory activity of benzothiazine derivatives **5**(**a-n**).

compound	substituent	urease inhibition IC_50_ ± s.e.m. (μM)
**5a**	-H	10.1 ± 0.90
**5b**	2-Methoxy	14.9 ± 1.50
**5c**	2-Methyl	17.2 ± 1.10
**5d**	2-Carboxymethyl	14.1 ± 0.12
**5e**	3-Methoxy	18.2 ± 1.69
**5f**	3-Methyl	15.5 ± 1.35
**5g**	4-Methoxy	20.4 ± 1.81
**5h**	4-Methyl	16.4 ± 1.40
**5i**	4-Chloro	20.7 ± 0.25
**5j**	4-Nitro	17.06 ± 1.45
**5k**	4-Carboxyethyl	9.8 ± 0.023
**5l**	4-Acetamido	12.6 ± 0.10
**5m**	2,3-Dichloro	14.06 ± 0.15
**5n**	2,4-Dichloro	13.06 ± 0.10
**thiourea**		22.3 ± 0.031

In parallel, the substitution of the methyl group on different positions of the phenylacetamide ring exhibited effective and comparable inhibitory potential for compounds **5c** (IC_50_ = 17.2 ± 1.10 µM), **5f** (IC_50_ = 15.5 ± 1.35 µM) and **5h** (IC_50_ = 16.4 ± 1.40 µM). Furthermore, the presence of the methoxy group on different positions of phenylacetamide ring in **5b**, **5e** and **5g** exhibited almost the same inhibitory potency as the IC_50_ values 14.9 ± 1.50 µM, 18.2 ± 1.69 µM and 20.4 ± 1.81 µM, respectively. Further modifications such as the entry of the nitro group (strong electron withdrawing) at *para*-position on phenylacetamide ring in **5j** showed better inhibition (IC_50_ value = 17.06 ± 1.45 µM) when compared with the standard drug.

### 2.3. Molecular docking studies

**Figure 2.**
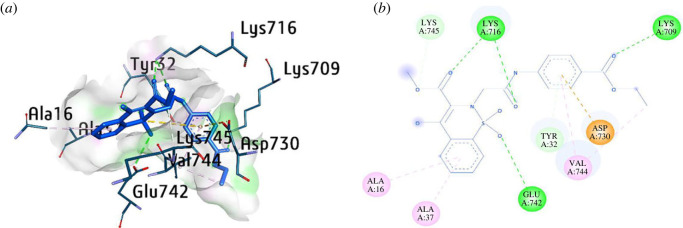
Three- and two-dimensional visualization of compound **5k** against urease.

The most potent compounds (**5a**, **5l** and **5k**) were docked within the active pocket of urease to elaborate important interactions with the amino acid residues of the receptor (PDB; 3LA4) [56]. The most potent compound **5k** presented various major interactions inside the urease active pocket with amino acid residues such as Lys709, Lys716, Lys745, Ala16, Ala37, Glu742, Val744, Tyr32 and Asp730. Conventional hydrogen bonds were observed with several amino acid residues including Lys716, Lys709 and Glu742 with the oxygen atom of thiazine derivative compound. π-Alkyl interactions were also observed with the benzothiazine ring with amino acid residues such as Ala16, Ala37 and Val744 with the phenylacetamide ring of compound **5k**. π-anion interactions were also exhibited in the phenylacetamide ring with Asp730, whereas Tyr32 and Lys745 were also found to interact with phenylacetamide ring and carboxylate part of the compound at distances of 4.10 Å and 3.60 Å, respectively (figure 2).

**Figure 3.**
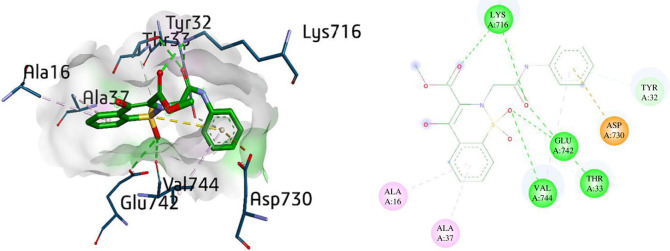
Three- and two-dimensional visualization of compound **5a** against urease.

The docking study of compound **5a** exhibited multiple interactions with amino acids such as Lys716 (2.80 Å and 2.89 Å), Glu742 (3.24 Å) and Thr33 (3.01 Å) depicting conventional hydrogen bond, with the oxygen atoms present in the benzothiazine compound. However, some other interactions like π-donor H-bond with Tyr32 (4.16 Å), π-anion with Asp730 (3.90 Å) and π-alkyl bond with Val744 (5.36 Å) were also observed in the phenylacetamide ring of the compound **5a**. Meanwhile, Ala16 (4.92 Å) and Ala37 (4.44 Å) formed π-alkyl interactions in the benzothiazine part of the compound. Moreover, Thr33 was also involved in the C–H bond with the phenyl ring via distance of 3.60 Å as shown in figure 3.

**Figure 4.**
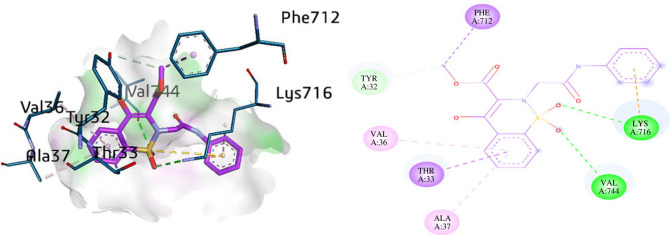
Three- and two-dimensional visualization of compound **5l** against urease.

Another potent inhibitor **5l** was docked inside the urease depicting various interactions with amino acid residues such as Phe712, Tyr32, Val36, Thr33, Ala37, Val744 and Lys716. Among them, Lys716 and Val744 showed conventional hydrogen bonding with the benzothiazine bearing oxygen atoms with distances of 3.11 Å and 3.24 Å, respectively. Lys716 also exhibited an π-cationic interaction with the phenylacetamide part of the compound **5l** via a distance of 4.64 Å. Additionally, π-sigma interactions were also observed between the amino acid residues such as Phe712 and Thr33 with the benzothiazine part of the compound. However, π-alkyl interactions were also revealed in the active moiety of the thiazine with amino acid residues Val36 and Ala37. Moreover, a C–H bond was established between carbon atom and hydroxyl group of the Tyr32 with a distance of 3.73 Å as shown in figure 4.

### 2.4. Molecular dynamic simulations

**Figure 5.**
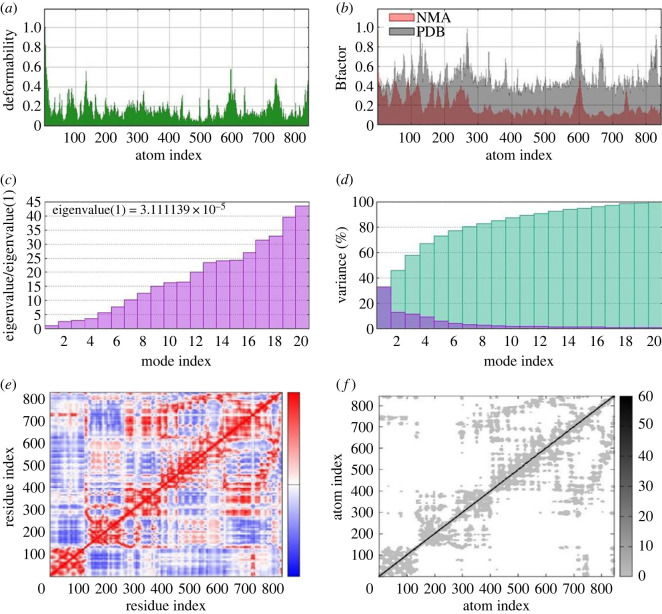
The molecular dynamic simulation study of docked complex **5k** with urease. Deformability (*a*), β-factor (*b*), eigen values (*c*), variance (*d*), covariance map (*e*) and elastic network (*f*). In (*d*), green colour indicates cumulative variances and red colour indicates individual variances, while in (*f*) darker grey area represents high stiff regions. In (*e*), covariance map represents correlated, anticorrelated or uncorrelated motions shown in red, blue or white colour, respectively.

The conformational dynamic studies of protein and compound **5k** complex were explored by employing molecular dynamic simulation through normal mode analysis (NMA) which is done by iMODS. Deformability results show a low level of deformation with all residues and the Eigen value of the complex is 3.111139 × 10^−5^. Figure 5 explains the results of MD simulation.

## 3. Experimental set-up

### 3.1. Synthesis

Chemicals acquired from E. Merck, Fluka or BDH were used as received, however solvents were distilled to purify. Gallenkamp melting point apparatus was used for determination of melting points of all the synthesized compounds. For FT-IR spectra, a Thermo Nicolet IR 200 spectrometer with KBr discs was used. A Brücker Avance NMR instrument was used for ^13^C NMR spectra at 75 MHz and ^1^H NMR spectra at 300 MHz. Mass spectra were recorded using the EI mode on a JEOL 600H-1 instrument. A LECO 630–200–200 TRUSPEC CHNS microanalyzer was used for elemental analysis and the data obtained was within ±0.4% of the calculated results. Compounds **2**–**4** were prepared according to the literature [55,57].

General synthetic procedure for 1,2-Benzothiazine-***N***-arylacetamides **5(a-n)**

To a solution of compound **3** (1.0 mmol) in 5 ml dry acetone was added anhydrous K_2_CO_3_ (1.5 mmol) and a solution of the corresponding 2-chloro-*N*-arylacetamide **4** (1.0 mmol) in 2 ml acetone drop-wise. The reaction mixture was refluxed for 6–8 h until the complete consumption of reactants as indicated by thin layer chromatography. Workup of the reaction was done by cooling, dilution with cold distilled water and acidification with cold dilute HCl (20%). The resulted precipitates were collected via filtration, washed with excess distilled water and dried at 70°C. Good to excellent isolated yields were recorded for all the products after re-crystallization with distilled methanol.

Methyl 4-hydroxy-2-(2-oxo-2-(phenylamino)ethyl)-2***H***-benzo[***e***][1,2]thiazine-3-carboxylate 1,1-dioxide **(5a)**

Yield 81%. White solid; m.p. 188–190°C. IR (KBr)cm^−1^: 3260 (N–H), 2951 (C–H), 1681 (C=O), 1602 (C=C), 1247 (C–N); ^1^H NMR (DMSO-*d_6_*, 300 MHz) *δ*: 3.91 (s, 3H, OC*H*_3_), 4.46 (s, 2H, C*H*_2_), 6.98 (t, *J* = 7.2 Hz, 1H, Ar*H*), 7.21 (t, *J* = 7.8 Hz, 2H, Ar*H*), 7.31 (d, *J* = 7.5 Hz, 2H, Ar*H*), 7.79–7.88 (m, 3H, Ar*H*), 8.04 (dd, *J* = 6.3 Hz, 2.1 Hz, 1H, Ar*H*), 9.98 (s, 1H, N*H*), 11.82 (s, 1H, O*H*) ppm; ^13^C NMR (DMSO-*d_6_*, 100 MHz) *δ*: 53.0, 53.5, 108.3, 119.4, 122.0, 123.8, 126.5, 128.2, 129.2, 132.8, 133.2, 138.3, 138.8, 158.0, 165.8, 168.7 ppm; Anal. calculated for C_18_H_16_N_2_O_6_S: C, 55.66; H, 4.15; N, 7.21; S, 8.26; Found: C, 55.70; H, 4.25; N, 7.29; S, 8.32; MS (EI+) *m/z*: 388.3 (M^+^).

Methyl 4-hydroxy-2-(2-((2-methoxyphenyl)amino)-2-oxoethyl)-2***H***-benzo[***e***][1,2]thiazine-3-carboxylate 1,1-dioxide **(5b)**

Yield 78%. Light yellow solid; m.p. 176–178°C. IR (KBr)cm^−1^: 3260 (N–H), 2950 (C–H), 1674 (C=O), 1605 (C=C), 1265 (C–N); ^1^H NMR (DMSO-*d_6_*, 300 MHz) *δ*: 3.83 (s, 3H, OC*H*_3_), 3.91 (s, 3H, OC*H*_3_), 4.42 (s, 2H, C*H*_2_), 6.79–6.85 (m, 1H, Ar*H*), 6.99–7.03 (m, 2H, Ar*H*), 7.75 (d, *J* = 7.8 Hz, 1H, Ar*H*), 7.83–7.90 (m, 3H, Ar*H*), 8.02–8.05 (m, 1H, Ar*H*), 9.32 (s, 1H, N*H*), 11.80 (s, 1H, O*H*) ppm; ^13^C NMR (DMSO-*d_6_*, 100 MHz) *δ*: 53.5, 53.8, 56.2, 108.9, 111.5, 120.7, 121.1, 122.6, 124.7, 126.7, 127.2, 128.2, 133.1, 133.3, 137.5, 149.3, 157.8, 166.1, 168.6 ppm; Anal. calculated for C_19_H_18_N_2_O_7_S: C, 54.54; H, 4.34; N, 6.70; S, 7.66; Found: C, 54.58; H, 4.36; N, 6.76; S, 7.70; MS (EI+) *m/z*: 418.3 (M^+^).

Methyl 4-hydroxy-2-(2-oxo-2-(***o***-tolylamino)ethyl)-2***H***-benzo[***e***][1,2]thiazine-3-carboxylate 1,1-dioxide **(5c)**

Yield 76%. White solid; m.p. 172–174°C. IR (KBr)cm^−1^: 3282 (N–H), 2959 (C–H), 1672 (C=O), 1609 (C=C), 1253 (C–N); ^1^H NMR (DMSO-*d_6_*, 300 MHz) *δ*: 2.04 (s, 3H, C*H*_3_), 3.93 (s, 3H, OC*H*_3_), 4.45 (s, 2H, C*H*_2_), 7.00–7.09 (m, 2H, Ar*H*), 7.13–7.16 (m, 2H, Ar*H*), 7.80–7.86 (m, 3H, Ar*H*), 8.00 (dd, *J* = 6.3 Hz, 1.2 Hz, 1H, Ar*H*), 9.41 (s, 1H, N*H*), 11.09 (s, 1H, O*H*) ppm; ^13^C NMR (DMSO-*d_6_*, 100 MHz) *δ*: 18.0, 53.0, 53.5, 108.4, 122.1, 125.0, 125.8, 126.4, 126.5, 128.3, 130.7, 132.0, 132.9, 133.2, 136.0, 138.0, 158.0, 165.8, 168.7 ppm; Anal. calculated for C_19_H_18_N_2_O_6_S: C, 56.71; H, 4.51; N, 6.96; S, 7.97; Found: C, 56.75; H, 4.55; N, 7.00; S, 8.03; MS (EI+) *m/z*: 402.1 (M^+^).

Methyl 4-hydroxy-2-(2-((2-(methoxycarbonyl)phenyl)amino)-2-oxoethyl)-2***H***-benzo[***e***][1,2]thiazine-3-carboxylate 1,1-dioxide **(5d)**

Yield 80%. White solid; m.p. 178–180°C. IR (KBr)cm^−1^: 3247 (N–H), 2959 (C–H), 1672 (C=O), 1590 (C=C), 1252 (C–N); ^1^H NMR (DMSO-*d_6_*, 300 MHz) *δ*: 3.84 (s, 3H, OC*H*_3_), 3.87 (s, 3H, OC*H*_3_), 4.34 (s, 2H, C*H*_2_), 7.19 (t, *J* = 7.8 Hz, 1H, Ar*H*), 7.56 (t, *J* = 7.8 Hz, 1H, Ar*H*), 7.87–7.92 (m, 4H, Ar*H*), 8.05 (d, *J* = 6.3 Hz, 1H, Ar*H*), 8.17 (d, *J* = 8.4 Hz, 1H, Ar*H*), 10.98 (s, 1H, N*H*), 11.83 (s, 1H, O*H*) ppm; ^13^C NMR (DMSO-*d_6_*, 100 MHz) *δ*: 53.0, 53.4, 54.4, 109.2, 118.2, 121.3, 123.0, 124.0, 126.8, 128.0, 131.1, 133.5, 134.4, 136.8, 139.3, 157.6, 166.7, 167.5, 168.5 ppm; Anal. calculated for C_20_H_18_N_2_O_8_S: C, 53.81; H, 4.06; N, 6.27; S, 7.18; Found: C, 53.97; H, 4.22; N, 6.43; S, 7.30; MS (EI+) *m/z*: 446.3 (M^+^).

Methyl 4-hydroxy-2-(2-((3-methoxyphenyl)amino)-2-oxoethyl)-2***H***-benzo[***e***][1,2]thiazine-3-carboxylate 1,1-dioxide **(5e)**

Yield 84%. White solid; m.p. 178–180°C. IR (KBr)cm^−1^: 3260 (N–H), 2950 (C–H), 1681 (C=O), 1674 (C=C), 1265 (C–N); ^1^H NMR (DMSO-*d_6_*, 300 MHz) *δ*: 3.65 (s, 3H, OC*H*_3_), 3.91 (s, 3H, OC*H*_3_), 4.43 (s, 2H, C*H*_2_), 6.58 (dd, *J* = 8.1 Hz, 1.8 Hz, 1H, Ar*H*), 6.88–6.93 (m, 2H, Ar*H*), 7.12 (t, *J* = 8.1 Hz, 1H, Ar*H*), 7.79–7.88 (m, 3H, Ar*H*), 8.04 (dd, *J* = 6.3 Hz, 2.1 Hz, 1H, Ar*H*), 9.98 (s, 1H, N*H*), 11.81 (s, 1H, O*H*) ppm; ^13^C NMR (DMSO-*d_6_*, 100 MHz) *δ*: 53.1, 53.5, 55.4, 105.5, 108.3, 109.0, 111.8, 122.0, 126.5, 128.3, 130.0, 132.8, 133.2, 138.2, 139.9, 158.0, 159.9, 165.8, 168.7 ppm; Anal. calculated for C_19_H_18_N_2_O_7_S: C, 54.54; H, 4.34; N, 6.70; S, 7.66; Found: C, 54.62; H, 4.42; N, 6.74; S, 7.68; MS (EI+) *m/z*: 418.2 (M^+^).

Methyl 4-hydroxy-2-(2-oxo-2-(***m***-tolylamino)ethyl)-2***H***-benzo[***e***][1,2]thiazine-3-carboxylate 1,1-dioxide **(5f)**

Yield 86%. Light brown solid; m.p. 192–194°C. IR (KBr)cm^−1^: 3254 (N–H), 2956 (C–H), 1670 (C=O), 1606 (C=C), 1253 (C–N); ^1^H NMR (DMSO-*d_6_*, 300 MHz) *δ*: 2.19 (s, 3H, C*H*_3_), 3.91 (s, 3H, OC*H*_3_), 4.44 (s, 2H, C*H*_2_), 6.81 (d, *J* = 3.6 Hz, 1H, Ar*H*), 7.09–7.11 (m, 3H, Ar*H*), 7.82–7.88 (m, 3H, Ar*H*), 8.04 (dd, *J* = 6.3 Hz, 1.8 Hz, 1H, Ar*H*), 9.91 (s, 1H, N*H*), 11.77 (s, 1H, O*H*) ppm; ^13^C NMR (DMSO-*d_6_*, 100 MHz) *δ*: 21.6, 53.0, 53.5, 108.2, 116.7, 119.9, 122.0, 124.6, 126.6, 128.2, 129.0, 132.8, 133.2, 138.2, 138.4, 138.7, 157.9, 165.7, 168.7 ppm; Anal. calculated for C_19_H_18_N_2_O_6_S: C, 56.71; H, 4.51; N, 6.96; S, 7.97; Found: C, 56.59; H, 4.47; N, 6.91; S, 7.90; MS (EI+) *m/z*: 402.3 (M^+^).

Methyl 4-hydroxy-2-(2-((4-methoxyphenyl)amino)-2-oxoethyl)-2***H***-benzo[***e***][1,2]thiazine-3-carboxylate 1,1-dioxide **(5g)**

Yield 86%. Light grey solid; m.p. 176–178°C. IR (KBr)cm^−1^: 3263 (N–H), 1672 (C=O), 1607 (C=C), 1249 (C–N); ^1^H NMR (DMSO-*d_6_*, 300 MHz) *δ*: 3.66 (s, 3H, OC*H*_3_), 3.91 (s, 3H, OC*H*_3_), 4.42 (s, 2H, C*H*_2_), 6.79 (d, *J* = 9.0 Hz, 2H, Ar*H*), 7.22 (d, *J* = 9.0 Hz, 2H, Ar*H*), 7.81–7.89 (m, 3H, Ar*H*), 8.03 (dd, *J* = 6.0 Hz, 2.4 Hz, 1H, Ar*H*), 9.87 (s, 1H, N*H*), 11.82 (s, 1H, O*H*) ppm; ^13^C NMR (DMSO-*d_6_*, 100 MHz) *δ*: 52.9, 53.5, 55.5, 108.2, 114.3, 121.0, 122.0, 126.5, 128.3, 131.9, 132.8, 133.2, 138.2, 155.7, 158.0, 165.2, 168.7 ppm; Anal. calculated for C_19_H_18_N_2_O_7_S: C, 54.54; H, 4.34; N, 6.70; S, 7.66; Found: C, 54.50; H, 4.28; N, 6.66; S, 7.58; MS (EI+) *m/z*: 418.3 (M^+^).

Methyl 4-hydroxy-2-(2-oxo-2-(***p***-tolylamino)ethyl)-2***H***-benzo[***e***][1,2]thiazine-3-carboxylate 1,1-dioxide **(5h)**

Yield 82%. White solid; m.p. 182–184°C. IR (KBr)cm^−1^: 3254 (N–H), 2956 (C–H), 1670 (C=O), 1606 (C=C), 1253 (C–N); ^1^H NMR (DMSO-*d_6_*, 300 MHz) *δ*: 2.19 (s, 3H, C*H*_3_), 3.91 (s, 3H, OC*H*_3_), 4.44 (s, 2H, C*H*_2_), 7.01 (d, *J* = 8.4 Hz, 2H, Ar*H*), 7.19 (d, *J* = 8.4 Hz, 2H, Ar*H*), 7.79–7.88 (m, 3H, Ar*H*), 8.03 (dd, *J* = 6.3 Hz, 2.4 Hz, 1H, Ar*H*), 9.89 (s, 1H, N*H*), 11.82 (s, 1H, O*H*) ppm; ^13^C NMR (DMSO-*d_6_*, 100 MHz) *δ*: 20.9, 53.0, 53.5, 108.3, 119.4, 122.0, 126.5, 128.2, 129.5, 132.1, 132.7, 133.2, 136.3, 138.3, 158.0, 165.5, 168.7 ppm; Anal. calculated for C_19_H_18_N_2_O_6_S: C, 56.71; H, 4.51; N, 6.96; S, 7.97; Found: C, 56.83; H, 4.65; N, 7.04; S, 8.03; MS (EI+) *m/z*: 402.3 (M^+^).

Methyl 2-(2-((4-chlorophenyl)amino)-2-oxoethyl)-4-hydroxy-2***H***-benzo[***e***][1,2]thiazine-3-carboxylate 1,1-dioxide **(5i)**

Yield 80%. White solid; m.p. 148–150°C. IR (KBr)cm^−1^: 3253 (N–H), 3111, 2954 (C–H), 1679 (C=O), 1598 (C=C), 1250 (C–N); ^1^H NMR (DMSO-*d_6_*, 300 MHz) *δ*: 3.90 (s, 3H, OC*H*_3_), 4.47 (s, 2H, C*H*_2_), 7.27 (dd, *J* = 6.9 Hz, 2.1 Hz, 2H, Ar*H*), 7.35 (dd, *J* = 6.9 Hz, 2.1 Hz, 2H, Ar*H*), 7.79–7.88 (m, 3H, Ar*H*), 8.04 (dd, *J* = 6.3 Hz, 2.1 Hz, 1H, Ar*H*), 10.16 (s, 1H, N*H*), 11.82 (s, 1H, O*H*) ppm; ^13^C NMR (DMSO-*d_6_*, 100 MHz) *δ*: 52.9, 53.5, 108.2, 120.9, 121.9, 126.5, 127.4, 128.2, 129.1, 132.8, 133.2, 137.8, 138.3, 158.0, 166.0, 168.7 ppm; Anal. calculated for C_18_H_15_ClN_2_O_6_S: C, 51.13; H, 3.58; N, 6.63; S, 7.58; Found: C, 51.19; H, 3.66; N, 6.71; S, 7.64; MS (EI+) *m/z*: 422.1 (M^+^).

Methyl 4-hydroxy-2-(2-((4-nitrophenyl)amino)-2-oxoethyl)-2***H***-benzo[***e***][1,2]thiazine-3-carboxylate 1,1-dioxide **(5j)**

Yield 85%. Brown solid; m.p. 192–194°C. IR (KBr)cm^−1^: 3349 (N–H), 3046 (C–H), 1646 (C=O), 1554 (C=C), 1286 (C–N); ^1^H NMR (DMSO-*d_6_*, 300 MHz) *δ*: 3.91 (s, 3H, OC*H*_3_), 4.56 (s, 2H, C*H*_2_), 7.56 (d, *J* = 9.3 Hz, 2H, Ar*H*), 7.81–7.91 (m, 3H, Ar*H*), 8.06 (dd, *J* = 6.3 Hz, 2.1 Hz, 1H, Ar*H*), 8.14 (d, *J* = 9.3 Hz, 2H, Ar*H*), 10.63 (s, 1H, N*H*), 11.83 (s, 1H, O*H*) ppm; ^13^C NMR (DMSO-*d_6_*, 100 MHz) *δ*: 53.0, 53.5, 108.2, 119.1, 121.9, 125.5, 126.6, 128.2, 132.9, 133.3, 138.2, 142.7, 144.9, 157.9, 167.0, 168.6 ppm; Anal. calculated for C_18_H_15_N_3_O_8_S: C, 49.88; H, 3.49; N, 9.70; S, 7.40; Found: C, 49.70; H, 3.31; N, 9.68; S, 7.32; MS (EI+) *m/z*: 433.3 (M^+^).

Methyl 2-(2-((4-(ethoxycarbonyl)phenyl)amino)-2-oxoethyl)-4-hydroxy-2***H***-benzo[***e***][1,2]thiazine-3-carboxylate 1,1-dioxide **(5k)**

Yield 82%. White solid; m.p. 208–210°C. IR (KBr)cm^−1^: 3364 (N–H), 2956 (C–H), 1664 (C=O), 1599 (C=C), 1254 (C–N); ^1^H NMR (DMSO-*d_6_*, 300 MHz) *δ*: 1.28 (t, *J* = 7.2 Hz, 3H, C*H*_3_), 3.90 (s, 3H, OC*H*_3_), 4.25 (q, *J* = 7.2 Hz, 2H, C*H*_2_), 4.51 (s, 2H, C*H*_2_), 7.46 (d, *J* = 9.0 Hz, 2H, Ar*H*), 7.82–7.88 (m, 5H, Ar*H*), 8.05 (dd, *J* = 6.3 Hz, 1.8 Hz, 1H, Ar*H*), 10.36 (s, 1H, N*H*), 11.82 (s, 1H, O*H*) ppm; ^13^C NMR (DMSO-*d_6_*, 100 MHz) *δ*: 14.6, 53.0, 53.5, 60.9, 108.2, 118.8, 122.0, 124.8, 126.6, 128.3, 130.7, 132.9, 133.2, 138.2, 143.1, 158.0, 165.7, 166.6, 168.7 ppm; Anal. calculated for C_21_H_20_N_2_O_8_S: C, 54.78; H, 4.38; N, 6.08; S, 6.96; Found: C, 54.90; H, 4.52; N, 6.20; S, 7.10; MS (EI+) *m/z*: 460.3 (M^+^).

Methyl 2-(2-((4-acetamidophenyl)amino)-2-oxoethyl)-4-hydroxy-2***H***-benzo[***e***][1,2]thiazine-3-carboxylate 1,1-dioxide **(5l)**

Yield 88%. White solid; m.p. 202–204°C. IR (KBr)cm^−1^ : 3402, 3277 (N–H), 3052, 2822 (C–H), 1671 (C=O), 1619 (C=C), 1252 (C–N); ^1^H NMR (DMSO-*d_6_*, 300 MHz) *δ*: 1.98 (s, 3H, C*H*_3_), 3.91 (s, 3H, OC*H*_3_), 4.43 (s, 2H, C*H*_2_), 7.22 (d, *J* = 8.7 Hz, 2H, Ar*H*), 7.41 (d, *J* = 8.7 Hz, 2H, Ar*H*), 7.79–7.89 (m, 3H, Ar*H*), 8.03 (dd, *J* = 6.3 Hz, 2.1 Hz, 1H, Ar*H*), 9.84 (s, 1H, N*H*), 9.93 (s, 1H, N*H*), 11.81 (s, 1H, O*H*) ppm; ^13^C NMR (DMSO-*d_6_*, 100 MHz) *δ*: 24.3, 52.9, 53.5, 108.2, 119.8, 122.0, 126.5, 128.2, 132.8, 133.2, 134.0, 135.4, 138.2, 158.0, 165.4, 168.4, 168.7 ppm; Anal. calculated for C_20_H_19_N_3_O_7_S: C, 53.93; H, 4.30; N, 9.43; S, 7.20; Found: C, 54.01; H, 4.36; N, 9.53; S, 7.32; MS (EI+) *m/z*: 445.3 (M^+^).

Methyl 2-(2-((2,3-dichlorophenyl)amino)-2-oxoethyl)-4-hydroxy-2***H***-benzo[***e***][1,2]thiazine-3-carboxylate 1,1-dioxide **(5m)**

Yield 85%. White solid; m.p. 154–156°C. IR (KBr)cm^−1^: 3267 (N–H), 2957 (C–H), 1681 (C=O), 1573 (C=C), 1247 (C–N); ^1^H NMR (DMSO-*d_6_*, 300 MHz) *δ*: 3.92 (s, 3H, OC*H*_3_), 4.51 (s, 2H, C*H*_2_), 7.27 (t, *J* = 8.1 Hz, 1H, Ar*H*), 7.41 (dd, *J* = 8.1 Hz, 1.5 Hz, 1H, Ar*H*), 7.54 (dd, *J* = 8.1 Hz, 1.5 Hz, 1H, Ar*H*), 7.82–7.87 (m, 3H, Ar*H*), 8.02 (dd, *J* = 6.0 Hz, 1.8 Hz, 1H, Ar*H*), 9.79 (s, 1H, N*H*), 11.80 (s, 1H, O*H*) ppm; ^13^C NMR (DMSO-*d_6_*, 100 MHz) *δ*: 53.1, 53.6, 108.5, 122.3, 123.9, 124.7, 126.6, 127.1, 128.2, 128.6, 132.3, 133.1, 133.3, 136.6, 137.7, 157.8, 166.6, 168.6 ppm; Anal. calculated for C_18_H_14_Cl_2_N_2_O_6_S: C, 47.28; H, 3.09; N, 6.13; S, 7.01; Found: C, 47.32; H, 3.11; N, 6.19; S, 7.13; MS (EI+) *m/z*: 456.1(M^+^).

Methyl 2-(2-((2,4-dichlorophenyl)amino)-2-oxoethyl)-4-hydroxy-2***H***-benzo[***e***][1,2]thiazine-3-carboxylate 1,1-dioxide **(5n)**

Yield 82%. White solid; m.p. 200–202°C. IR (KBr)cm^−1^: 3364 (N–H), 2957 (C–H), 1663 (C=O), 1581 (C=C), 1246 (C–N); ^1^H NMR (DMSO-*d_6_*, 300 MHz) *δ*: 3.92 (s, 3H, OC*H*_3_), 4.50 (s, 2H, C*H*_2_), 7.21 (dd, *J* = 8.7 Hz, 2.4 Hz, 1H, Ar*H*), 7.50 (d, *J* = 8.7 Hz, 1H, Ar*H*), 7.69 (d, *J* = 2.4 Hz, 1H, Ar*H*), 7.84–7.90 (m, 3H, Ar*H*), 8.04 (dd, *J* = 6.0 Hz, 2.4 Hz, 1H, Ar*H*), 9.72 (s, 1H, N*H*), 11.79 (s, 1H, O*H*) ppm; ^13^C NMR (DMSO-*d_6_*, 100 MHz) *δ*: 53.2, 53.6, 108.6, 122.5, 124.0, 126.1, 126.7, 128.2, 131.3, 132.0, 133.2, 133.4, 135.9, 137.6, 157.7, 166.9, 168.5 ppm; Anal. calculated for C_18_H_14_Cl_2_N_2_O_6_S: C, 47.28; H, 3.09; N, 6.13; S, 7.01; Found: C, 47.36; H, 3.21; N, 6.25; S, 7.13; MS (EI+) *m/z*: 456.1 (M^+^).

### 3.2. Urease inhibition activity assay

Compounds **5(a-n)** were investigated to evaluate the inhibitory activity employing the slightly modified indophenol method [58,59]. Assay mixture containing 1 unit of urease enzyme solution and 60 µl of buffer comprising urea substrate (100 mM) were pre-incubated with synthesized compounds (10 µl of 1 mM solution) at room temperature for 10 min in 96-well plates. Inhibitory activity of all test compounds was noted by measuring NH_3_ production using the above-mentioned protocol. Momentarily, phenolic reagent (50 µl) and alkali reagent (70 µl) were added in each well. After half an hour, absorbance at 630 nm was determined for all, using a Bio-Tek ELx 800, Instruments, Inc. USA Elisa-plate reader. Triplicate data for all the experiments was obtained and percentage inhibitory activities were calculated using the following formula:

$$\mathrm{Inhibition}\;\mathrm{percentage}\;=100-\left(\;\frac{\mathrm{absorbance}\;\mathrm{of}\;\mathrm{experimental}\;\mathrm{compound}}{\mathrm{absorbance}\;\mathrm{of}\;\mathrm{test}\;\mathrm{control}}\right)\times 100.$$

### 3.3. Docking protocol

#### 3.3.1. Structure assortment and preparation

Using the molecular docking protocol, the jack bean urease's crystallographic structure (3LA4; PDB) was downloaded from the protein data bank library [56] and prepared for the investigation by forming the complex with urease enzyme. Before docking analysis, structures of compounds and enzyme were prepared. To protonate the structure of the enzyme within the molecular modelling software MOE, the protonate3D technique was used [60,61]. Structure energy of molecules as well as molecules of solvent was minimized using the Amber99 force field. To overcome the collapsing of binding pockets during the calculations of minimization of energy, small force was used to strengthen the backbone atoms. Consequently, the ligands as well as water molecules were removed and addition of polar hydrogens was carried out in the crystallographic structure employing MOE.

#### 3.3.2. Compounds preparation

A ‘wash' module was applied to the three-dimensional structural coordinates of synthesized compounds **(5a, 5l and 5k)** for the assignment of protonation process and ionization state within the specific range of pH by MOE. Subsequently, for the investigation of docking, the energy of the chemical structure was minimized using the MMFF94x force field.

#### 3.3.3. Docking studies

LeadIT software from BioSolveIT, GmbH Germany [62] was used for calculations and docking investigations. The LeadIT tool, receptor utility was uploaded by Load or Prepare Receptor tool along with metal ions identified as part of it. The binding site was defined within the 9.0 spacing of residues. Docking of the inhibitor was performed employing the FlexX utility from LeadIT. Depending upon the free binding energies for all the three compounds **5a, 5l** and **5k**, almost 50 different conformations for each complex were collected. Already defined parameters of docking were used, and the best 30 poses were selected for further analysis [63]. Poses having less binding free energy were selected as the stable and exhibited maximum affinity with the load receptor. Such three-dimensional models were fabricated using Discovery Studio Visualization v4 to evaluate the interactions between ligand–protein complex having minimum free binding energy [64].

#### 3.3.4. Molecular dynamics simulations

These were performed by online server iMODs for the docking investigation as well as the dynamics of the complex to determine the motion of molecules. Normal Mode Analysis (NMA) was also determined through the iMODs server [65]. This tool also generates different parameters such as deformability, B-factors, eigenvalue, variance, covariance and elastic network. For these investigations, a PDB file was used to upload the input file on the iMODs server.

## 4. Conclusion

In summary, a series of new benzothiazine **5**(**a-n**) derivative compounds were designed and synthesized through a multistep and facile approach, considering the significance of thiazine as a versatile category of new drugs for the treatment of ureolytic infections. Substitution of electron deficient or rich groups at different positions of the phenyl ring provided a huge opportunity for demonstrating important interactions with different amino acid residues. All the compounds **5**(**a-n**) exhibited outstanding inhibitory potential (9.8–20.7 µM) when compared with positive control thiourea (22.3 ± 0.031), among which **5k** emerged as the best candidate with strong inhibition depicting an IC_50_ value (9.8 ± 0.023 µM). To identify the important parameters required to increase potency, structure–activity relationship was generated. An *in silico* study showed the conventional hydrogen bonding, π-sigma, π-alkyl as well as π-cation interactions with different amino acid residues like Lys716, Phe712, Ala16 and Val744. Molecular dynamics simulations validated the stability and deformability of the protein with the inhibitors. Therefore, a new template based on the identified inhibitors could be developed for the treatment of ureolytic bacterial infections using the identified inhibitors.

## Data Availability

The data presented in this study are available in electronic supplementary material which include NMR and mass spectra of all the synthesized compounds, SeeSAR visual drug design and ADMET Properties of the synthesized compounds [66].
